# Neural functions in cancer: Data analyses and database construction

**DOI:** 10.3389/fgene.2023.1062052

**Published:** 2023-02-13

**Authors:** Renbo Tan, Feilong Wang, Yi Zhou, Zhenyu Huang, Zheng An, Ying Xu

**Affiliations:** ^1^ Key Laboratory of Symbolic Computation and knowledge Engineering, College of Computer Science and Technology, Jilin University, Changchun, China; ^2^ Cancer Systems Biology Center, China-Japan Union Hospital of Jilin University, Changchun, China; ^3^ Computational Systems Biology Lab, Department of Biochemistry and Molecular Biology, And Institute of Bioinformatics, University of Georgia, Athens, GA, United States

**Keywords:** neural functions, pan-cancer, metastasis, transcriptomic data, database

## Abstract

Recent studies have revealed that neural functions are involved in possibly every aspect of a cancer development, serving as bridges connecting microenvironmental stressors, activities of intracellular subsystems, and cell survival. Elucidation of the functional roles played by the neural system could provide the missing links in developing a systems-level understanding of cancer biology. However, the existing information is highly fragmented and scattered across the literature and internet databases, making it difficult for cancer researchers to use. We have conducted computational analyses of transcriptomic data of cancer tissues in TCGA and tissues of healthy organs in GTEx, aiming to demonstrate how the functional roles by the neural genes could be derived and what non-neural functions they are associated with, across different stages of 26 cancer types. Several novel discoveries are made, including i) the expressions of certain neural genes can predict the prognosis of a cancer patient; ii) cancer metastasis tends to involve specific neural functions; iii) cancers of low survival rates involve more neural interactions than those with high survival rates; iv) more malignant cancers involve more complex neural functions; and v) neural functions are probably induced to alleviate stresses and help the associated cancer cells to survive. A database, called NGC, is developed for organizing such derived neural functions and associations, along with gene expressions and functional annotations collected from public databases, aiming to provide an integrated and publicly available information resource to enable cancer researchers to take full advantage of the relevant information in their research, facilitated by tools provided by NGC.

## 1 Introduction

Cancer tissue-based studies have revealed that the nervous system plays essential roles in cancer formation, progression, and metastasis (Wang et al., 2021). Such roles range from neuroinflammatory activities at the onset of a cancer, the guiding roles of neural projection throughout cancer development to the recently discovered driving roles of Schwann cells in cancer migration and metastasis (Deborde and Wong, 2017). Cross-talks have been widely observed between cancer and neural cells, such as neurites growing towards cancer, referred to as neo-neurogenesis, and cancer cells invading nerves, called perineural invasion, which can regulate the inflammatory states in the cancer-forming microenvironment (Yoneda et al., 2018). Neurotransmitters have been found to affect the activities of immune cells in the cancerous microenvironment by modulating cancer vascularization, invasion, and metastasis (Tilan and Kitlinska, 2010; Li and Cho, 2011; Jiang et al., 2020). Both the sympathetic and parasympathetic nerves are known to play key roles in cancer development and metastasis as in prostate cancer (March et al., 2020). Similar has been observed about the vagal nerve (De Couck et al., 2018).

Considerable information has also been generated about the interactions between neural and non-neural functions in cancer. For example, it has been reported that adrenergic innervation could act on T cells, leading to T cell-induced ACh and also regulating the local macrophages (Reardon et al., 2013). Damage of perineurium by invading cancer cells can trigger a cascade of inflammatory cytokines, which further induces axon regeneration to guide cancer tumor growth (Wang et al., 2021).

The increasing pool of information about the strong interactions between cancer and the nerve system, both local and distal, has not only expanded our knowledge of cancer as a whole-body level disease but also suggested novel ways for cancer treatment. For example, breast cancer treatment *via* denervation *via* blocking neurotransmission has generated promising results (Kappos et al., 2018). Similar studies have been reported on other cancers including brain, pancreas, prostate, skin, and stomach cancers (Denmeade and Isaacs, 2002).

Knowing the strong relevance of neo-neurogenesis to cancer development and treatment, we expect that increasingly more studies on detailed relationships between cancer and neural cells will emerge. Hence, we see an increasing need for elucidating the basic functional roles of neural genes throughout cancer development in a systematic manner and for making such information publicly available.

To demonstrate the feasibility in deriving such information in a systematic manner through analyses of cancer tissue-based transcriptomic data and the effectiveness in applying it to elucidation of cancer biology, we have conducted a preliminary study of functional roles of all human neural genes in cancer tissues in the TCGA database. Our findings suggest that i) the expressions of certain neural genes have strong impacts on the survival of cancer patients; ii) a set of neural genes can be identified in each cancer type, whose expressions are associated with cancer metastasis; iii) cancer patients with low survival rates generally involve more interactions with neural functions, compared to those with higher survival rates; iv) more generally, more complex neural functions are required in more malignant cancers; and v) neural genes play key roles in helping cancer cells survive the local environmental stressors.

Based on this study, we have developed a database, named *Neural Genes in Cancer* or *NGC*, containing the functional information of neural genes in multiple cancer types and stages, which we consider as highly relevant to gaining improved understanding of cancer biology. Currently, NGC covers 26 cancer types, namely, adrenocortical carcinoma (ACC), bladder urothelial carcinoma (BLCA), breast invasive carcinoma (BRCA), cervical squamous cell carcinoma and endocervical adenocarcinoma (CESC), colon adenocarcinoma (COAD), lymphoid neoplasm diffuse large B-cell lymphoma (DLBC), esophageal carcinoma (ESCA), glioblastoma multiforme (GBM), kidney chromophobe (KICH), kidney renal clear cell carcinoma (KIRC), kidney renal papillary cell carcinoma (KIRP), brain lower grade glioma (LGG), liver hepatocellular carcinoma (LIHC), lung adenocarcinoma (LUAD), lung squamous cell carcinoma (LUSC), ovarian serous cystadenocarcinoma (OV), pancreatic adenocarcinoma (PAAD), prostate adenocarcinoma (PRAD), rectum adenocarcinoma (READ), skin cutaneous melanoma (SKCM), stomach adenocarcinoma (STAD), testicular germ cell tumors (TGCT), thyroid carcinoma (THCA), thymoma (THYM), uterine corpus endometrial carcinoma (UCEC), and uterine carcinosarcoma (UCS), along with transcriptomic data of normal tissues of 20 relevant organs from the GTEx database as controls (Ardlie et al., 2015). These cancer types represent all the types in TCGA with sufficiently large numbers of tissue samples needed by our analyses. In addition, NGC also provides a suite of interactive tools for a) differential gene expression analyses between cancer and control tissues, b) survival analyses of given genes and cancer, c) pathway enrichment analyses of specified genes and cancer, d) co-expression analyses between neural and non-neural genes related to specified functions in cancer, such as metastasis, and e) weighted correlation network analyses (WGCNA) between neural genes. Furthermore, it provides free downloads of the analysis results *via* a simple interface.

Based on the new insights gained through our preliminary analyses, we anticipate that NGC will serve as a powerful tool to cancer researchers, regardless of their computer programming skills, to perform functional analyses of neural genes in cancer. The database is freely available at http://csbl.bmb.uga.edu/NGC.

## 2 Materials and methods

### 2.1 Differential gene expression analyses

A basic premise in conducting our study, as in many similar studies, is that only differentially expressed genes in disease vs. control tissues are considered as relevant to the occurrence and development of the disease. Hence only such genes are included when analyzing transcriptomic data of each cancer type. Normalized raw counts from TCGA and GTEx are downloaded and used in our study; and DESeq2 is used to assess differentially expressed neural genes in tissue samples of each cancer type/stage vs. controls (Love et al., 2014). Genes with |log_2_FC| > 1 and q-value <0.05 are considered as differentially expressed, where FC is for fold change between the average expression of a specified gene across all the cancer samples under consideration vs. controls.

### 2.2 Cox regression analyses

Both the univariate and multivariate Cox regression analyses are employed for survival analyses, using the “survival” and “survminer” packages in R (Kassambara et al., 2021; Therneau et al., 2022), respectively, representing two most widely used techniques for survival analyses. Specifically, all expressed neural genes are individually examined to check if its expression level has statistically significant implications to the survival rate, determined using the univariate Cox regression analysis. A multivariate Cox regression is then conducted to predict the prognosis based on all the selected genes for each cancer type, hence the two analyses serving different purposes. We have screened neural genes having *p*-value <0.05 from the univariate Cox regression analyses as the candidate genes, and then used a Lasso penalty to further screen neural genes for multivariate Cox regression analyses that keeps the number of selected genes minimal without losing the predictive power. Kaplan-Meier, Cox, and ROC analysis are used to validate the regression model on a validation dataset, and the neural gene set achieving AUC > 0.7 and *p*-value <0.05 is selected as the final prognostic markers, where the Kaplan-Meier survival estimate is a univariable analysis method for estimating the survival probability from the observed survival times (Rich et al., 2010); and AUC (Area Under Curve) is defined as the area enclosed by the coordinate axis under the receiver operating characteristic (ROC) curve. In the univariate or multivariate Cox analyses, hazard ratio (HR) is the ratio between the death and live rates reflected by the expression of a survival-related gene, as shown in Supplementary Tables S1–S3, where HR < 1 means that the increased expression of the gene improves survival while HR > 1 means the expression reduces survival.

### 2.3 Sample classification by random forest

The random forest method is employed to identify genes whose expressions collectively distinguish among samples with distinct labels, which represents a widely used technique for classification problems (Fawagreh et al., 2014; More and Rana, 2017; Sarica et al., 2017). Here, we consider samples with two distinct labels, metastatic and non-metastatic cancers. We identify neural gene biomarkers of metastasis in ten cancers: ACC, BLCA, BRCA, COAD, KIRC, KIRP, LUAD, READ, STAD, and THCA, representing all the TCGA cancer types each having at least ten stage-IV samples required by our analyses, with the detailed information given in Supplementary Table S4. We consider tissues of cancer stages I and II as the non-metastatic group, stage IV as the metastatic group (NOTE: stage III is not used since some stage-III samples have lymph node spread while others do not). The random forest with feature selection is used to conduct the classification analysis (Menze et al., 2009). Specifically, the randomForest() function in the randomForest package of R was used to conduct the random forest-based analyses, and the Gini index (Menze et al., 2009) was employed to construct a decision tree. A 10-fold cross-validation method is used to evaluate the classification performance.

### 2.4 Correlation analysis

The Pearson correlation coefficient (PCC), a classic method for measuring linear correlation between two data sets, is calculated between the expressions of two specified lists of genes. In this study, a gene pair with PCC >0.8 and *p*-value <0.05 is considered as a co-expressed gene pair.

### 2.5 Pathway enrichment analysis

ClusterProfiler in the R package is used for pathway enrichment analyses against the Gene Ontology (GO) database in this study, which employs both over-representation analysis (ORA) and gene set enrichment analysis (GSEA) techniques and represents a highly used package for pathway enrichment analyses (Wu et al., 2021).

### 2.6 Clustering analyses based on Co-expressed genes

For a given set of genes and their expressions in a specified set of tissues, their pairwise co-expressions are calculated using the “WGCNA” package in R (Langfelder and Horvath, 2008), a most widely used package for gene co-expression analyses, from which clusters of strongly co-expressed genes are identified. Specifically, an adjacency matrix was constructed based on the weighted correlation matrix among the genes, and the adjacency matrix was transformed into a Topology Overlap Matrix (Tian et al., 2020), from which a hierarchical clustering is conducted using the dynamicTreeCut algorithm (Langfelder et al., 2008).

## 3 Results

### 3.1 Neural genes expressions can predict cancer patients’ survival

To study the possible impact of neural genes on cancer patients’ survival, we have identified all neural genes expressed in cancer tissues and examined their expression profiles across each of the 26 cancer types. By comparing the numbers of upregulated and downregulated neural genes across different cancer types, we note: thirteen cancer types each have more upregulated neural genes than the number of downregulated ones (Supplementary Figure S1A). Five cancer types each have comparable numbers of up- and downregulated neural genes (Supplementary Figure S1B). And the remaining eight each have more downregulated neural genes than the upregulated ones (Supplementary Figure S1C). These data reveal that different cancer types have distinct levels of involvement of neural functions. Supplementary Table S5 lists differentially expressed neural genes along with fold changes and *p*-values across the 26 cancer types.

**FIGURE 1:**
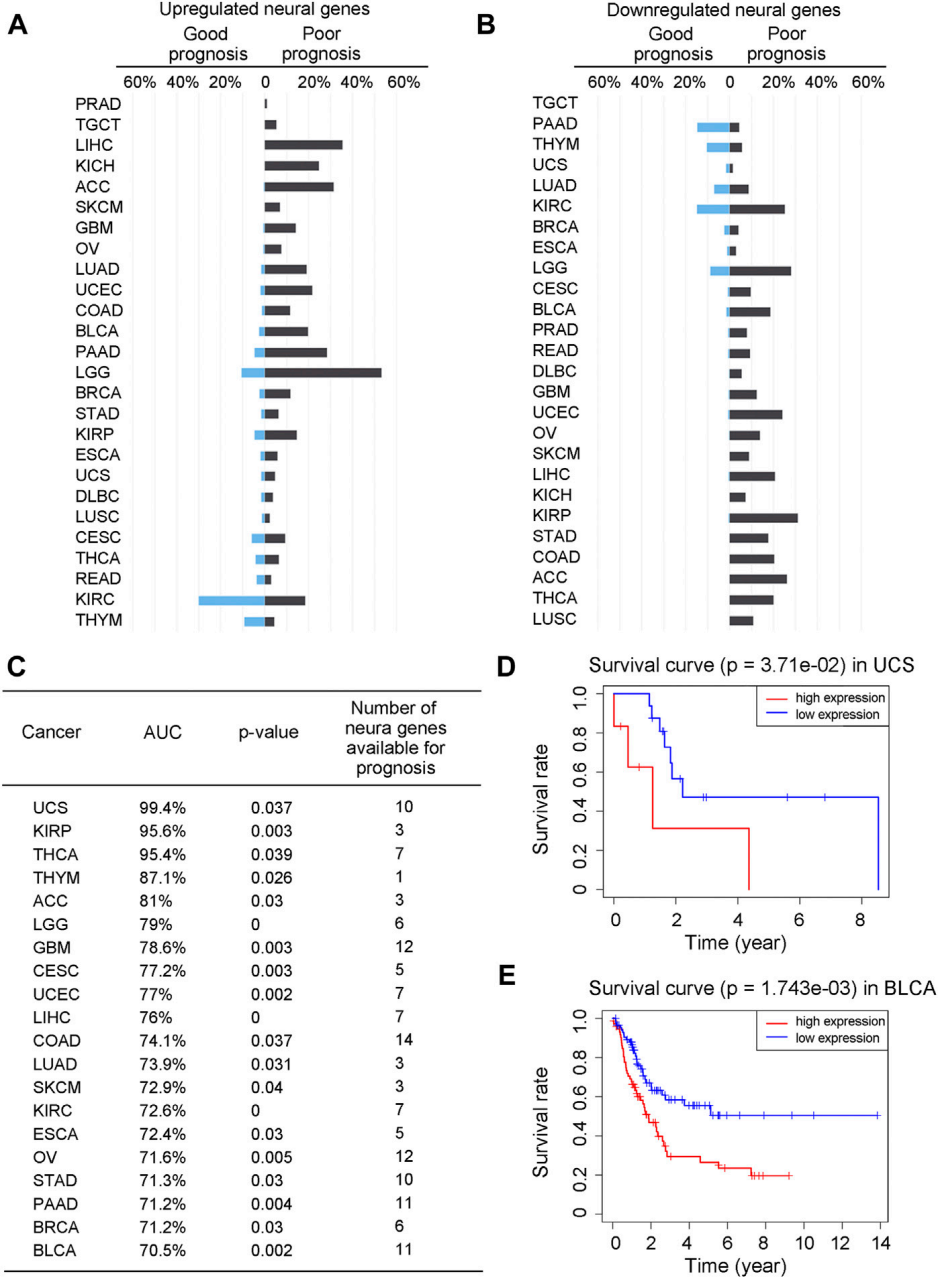
The expressions of neural genes can predict prognosis. **(A, B)** Bar graphs for the proportions of upregulated neural genes with good prognosis (HR < 1 and *p*-value <0.05) or poor prognosis (HR > 1 and *p*-value <0.05) across 26 cancer types **(A)**, and downregulated neural genes with good or poor prognosis **(B)**. Genes in blue are for good prognosis, while genes in black are for poor prognosis. **(C)** Prediction result of the Cox regression model across 20 cancer types, with AUC, *p*-value, and the number of selected prognostic neural marker genes. **(D, E)** Kaplan-Meier curves and the *p*-values for UCS and BLCA over the test sets of the Cox regression model, respectively. The x-axis is age in years and the y-axis is the survival rate. Gene expressions greater than the median is considered as high expressions, otherwise low expressions.

To understand how neural functions contribute to cancer development, we have first examined the contribution of the neural genes to the prognosis of cancer patients. Specifically, we have conducted a univariate Cox regression analysis against the expressions of the differentially expressed neural genes (see METHODS) and calculated the hazard ratio (HR, the ratio between the rates for death and for live) of each neural gene. For each cancer type, we have calculated the proportion of its neural genes with good prognosis (HR < 1 with *p*-value <0.05) and that with poor prognosis (HR > 1 with *p*-value <0.05) among all the differentially expressed neural genes, respectively. The calculation results for each of the 26 cancer types are given in Supplementary Tables S1—S2 and summarized in Figures 1A, B, from which we can see that more upregulated neural genes have poor than good prognosis in 23 out of the 26 cancers (except for READ, KIRC and THYM), and more downregulated neural genes have poor than good prognosis in 22 out of the 26 cancers (except for PAAD, TGCT, THYM and UCS), revealing that more differentially expressed neural genes, up- or downregulated, imply poorer survival in general.

A natural question is: can we predict the prognosis of a patient based on the expressions of neural genes? To answer the question, we have conducted a Cox regression analysis (with LASSO penalty) of the five-year survival rates of the patients (as provided in TCGA) of a cancer type against the expressions of selected neural genes, namely, to select neural genes whose expressions can collectively well explain the survival data for all cancer samples of each cancer type (see METHODS). Considering that five cancer types, namely, DLBC, KICH, PRAD, READ, and TGCT, each have very limited survival data in TCGA, our analyses are conducted on the other 21 cancer types.

The detailed analysis results are given in Supplementary Table S3 with a summary shown in Figure 1C. We note that of the 21 cancer types, 20 each have its survival rates well explained by a set of cancer type-specific neural genes. Specifically, UCS has the highest explainability of the survival rates by the selected neural genes, achieving 99.4% while BLCA has the lowest one at 70.5%, measured using the Kaplan-Meier score (see METHODS). Survival curves along with *p*-values for UCS and BLCA are shown in Figures 1D, E as examples. It is noteworthy that the survival rates of LUSC could not be well explained by its neural genes. One possible explanation is that LUSC is known to be predominantly associated with cigarette smoking, which may give rise to a distinct determinant for the cancer’s malignancy level compared to the other cancer types. Interestingly, for each of the 20 cancer types, at least 120 of the selected survival neural genes have been reported to be critical to the survival of the cancer type as listed in Supplementary Table S6, hence strongly supporting our predictions.

### 3.2 Neural genes and cancer metastasis

We have examined how the number of upregulated neural genes changes with the progression of a cancer from stage I through stage IV and noted that this number generally increases with the disease progression, particularly at stage IV (Supplementary Figures S2—S3), suggesting that more malignant cancers require more neural genes and furthermore, metastasis involves neural genes.

**FIGURE 2:**
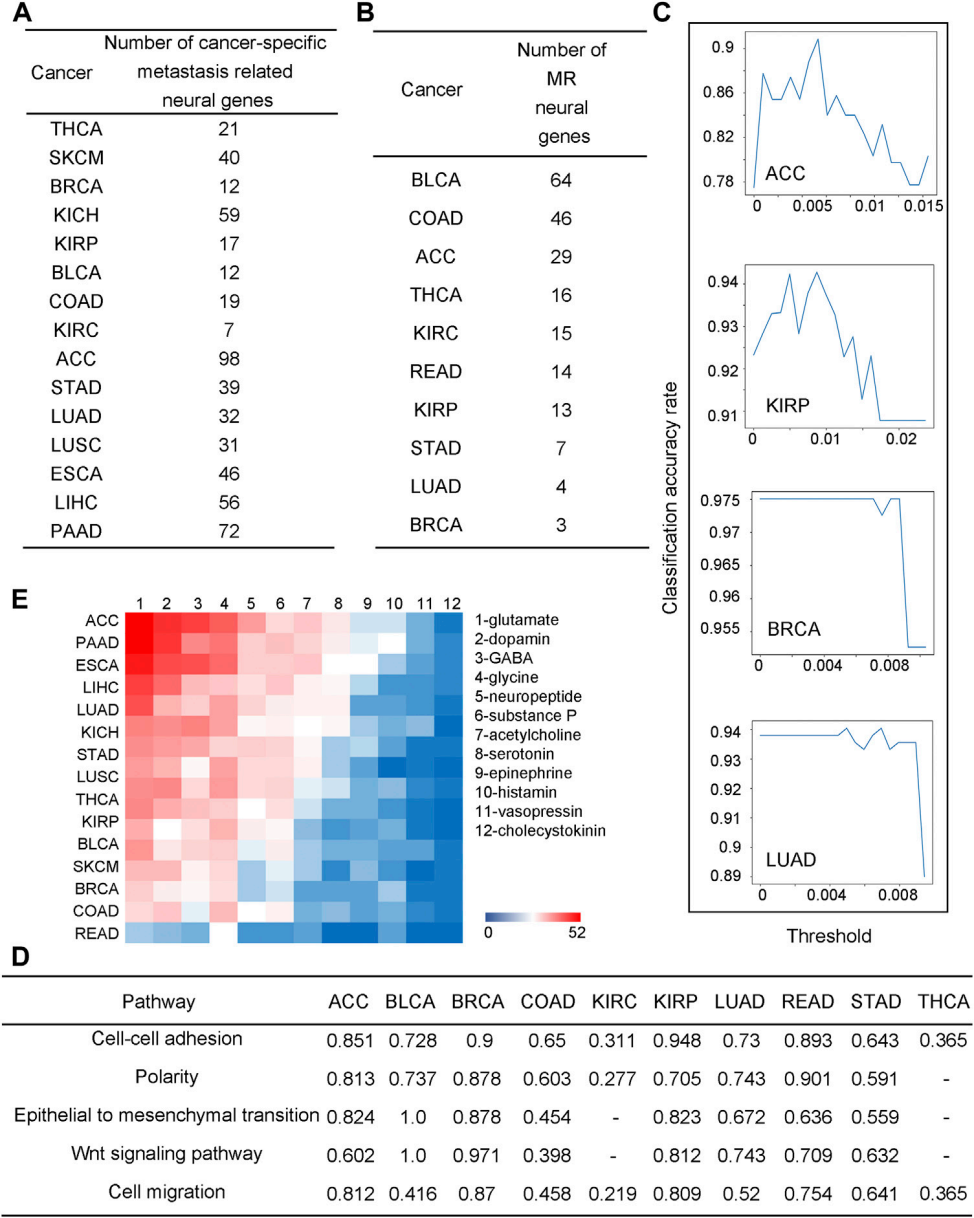
Neural genes are involved in cancer metastasis. **(A)** The number of neural genes strongly co-expressed with metastatic genes across different cancers. **(B)** The number of neural genes selected by the random forest-based classification. **(C)** Line charts for the classification performance by the random forest model for ACC, BRCA, KIRP, and LUAD. The x-axis is the threshold for feature selection, where if the value of a feature is lower than the threshold, the feature will be deleted, and the y-axis is for the classification accuracy. **(D)** Correlation coefficients between selected neural genes and metastatic pathways, “-” is for coefficients not statistically significant, i.e., *p*-value >0.05. **(E)** A heatmap for co-expressions between neurotransmitter genes and metastatic genes.

To investigate their detailed functional roles in metastasis, correlation analyses are performed between the upregulated neural genes and 2,091 metastasis-related genes across all stage IV cancer tissues for each of the 26 cancer types, collected from the human cancer metastasis database (Zheng et al., 2018), metastasis related genes in uniport (Consortium UniProt, 2021) and our previous study (Sun et al., 2020) (see METHODS and Supplementary Table S7). The number of upregulated neural genes whose expressions strongly correlate with metastasis genes (coefficients ≥ 0.8 with *p*-value <0.05) is summarized in Figure 2A for each cancer type. ACC has an average 5-year survival rate at ∼50%. A cancer type with the survival rate higher than ACC is considered as having a high survival rate; and similarly, a cancer with the survival rate lower than ACC as having a low survival rate. We note that the lower the (average) survival rate a cancer type has, the more upregulated neural genes co-expressed with metastatic genes. It is noteworthy that downregulated neural genes do not have a similar pattern.

Now we check if some neural genes can be used as metastasis biomarkers. To address this issue, we consider cancer tissues in stages I and II as non-metastatic and stage IV samples as metastatic cancers. We have conducted a classification analysis using the expressions of the to-be-selected upregulated neural genes to distinguish metastatic from non-metastatic cancer tissues over samples of each of the following ten cancer types: ACC, BLCA, BRCA, COAD, KIRC, KIRP, LUAD, READ, STAD, and THCA, representing all the cancer types each having at least ten stage IV samples in TCGA. A random forest approach is used to conduct the classification analyses (see METHODS). For each cancer type, the selected neural genes are subject to a 10-fold cross-validation.

We note that for each cancer type, a varying number of upregulated neural genes is needed to distinguish the metastatic from the non-metastatic cancer tissues, summarized in Figure 2B, ranging from three (BRCA) to 64 (BLCA), which gives somewhat different classification accuracies across different cancer types, from 0.975 (BRCA) to 0.736 (BLCA) (see METHODS), as shown in Figure 2C; Supplementary Figure S4. Each set of the selected neural genes is considered as metastatic markers (MR) for the cancer type. Literature review provided strong supporting evidence to our selected MRs for each cancer type (see Supplementary Table S8).

For each cancer type, a pathway enrichment analysis is conducted over all the expressed metastasis-related genes (see METHODS). For each enriched pathway, a Principal Component Analysis (PCA) is conducted on the gene set enriching the pathway. Its first principal component is used as the representative of the gene set. Similarly, a PCA is also conducted on the MR genes for each cancer type and a representative is selected. Subsequentially, a correlation analysis is conducted between these two representatives. The calculation results, shown in Figure 2D, reveal that the MR genes highly correlate with the metastasis-related functions, hence indicating that such MRs could be reliably used as metastatic markers.

Previous studies suggest that neurotransmitters secreted by nerves are key to cancer metastasis (Jobling et al., 2015). Here, we have studied the expressions of all major neurotransmitters: glutamate, dopamine, GABA, glycine, neuropeptide, substance *p*, acetylcholine, serotonin, epinephrine, histamine, vasopressin, and cholecystokinin by calculating the Pearson correlation coefficients (PCCs) between the metastatic genes and each neurotransmitter-synthesizing gene. Figure 2E shows the correlation coefficients as a heatmap with cancers ordered, from the highest to the lowest, by their numbers of neurotransmitters that highly correlate with metastatic genes. We note that glutamate, an excitatory neurotransmitter, is the mostly used among all the 12 neurotransmitters, which is consistent with a previous study (Stepulak et al., 2014). Interestingly, the average survival rate for eight cancer types having the highest number of metastasis-correlated neurotransmitters is 33%, while that for the seven cancer types having the lowest such neurotransmitters is 86%, consistent with the previous study (Ries et al., 2008). Further details are given in Supplementary Table S9.

### 3.3 Contributions of neural genes to cancer development

#### 3.3.1 Functional analyses of differentially expressed neural genes across 26 cancer types

Pathway enrichment analyses are conducted over all up- and downregulated neural genes, respectively, to study the neural functions that contribute to the development of each of the 26 cancer types. Supplementary Tables S10, S11 list the pathways enriched by the up- and downregulated neural genes, respectively, across the 26 cancer types.

**FIGURE 3:**
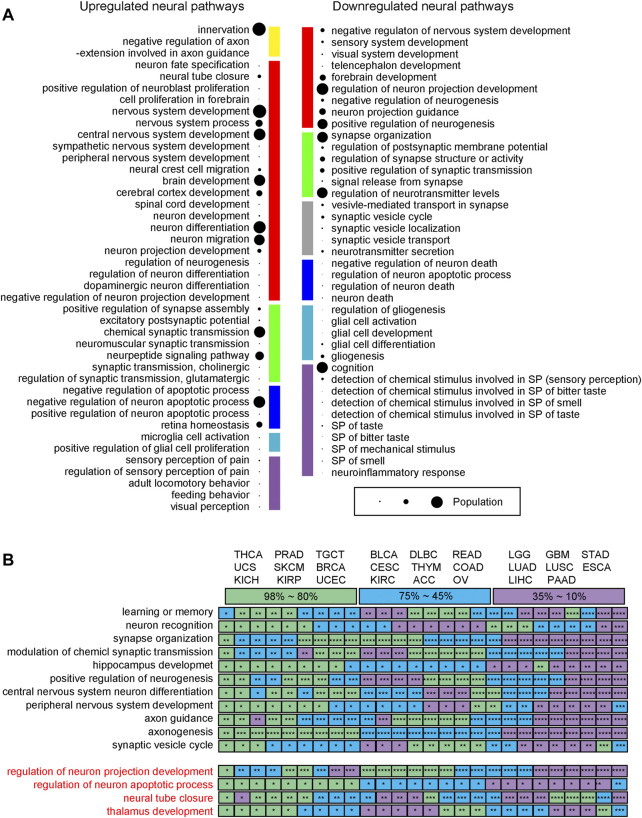
Neural functions across 26 cancer types. **(A)** Functions of upregulated and downregulated neural genes. The size of a bubble represents the number of pathways covered, ranging from 1 to 26. Yellow is for axon guidance related pathways. Red is for neuron differentiation related pathways. Green is for synapse organization related pathways. Blue is for neuron apoptosis related pathways. Sky blue is for glia cell activation related pathways. Purple is for neuronal perception related pathway; and grey denotes neurotransmitter transport related pathways. **(B)** The mean five-year survival rates for different cancer types according to the SEER reports, where the rates are color coded with purple for 10%–35% covering ESCA, GBM, LGG, LIHC, LUAD, LUSC, PAAD, and STAD, blue for 45%–75% covering ACC, BLCA, CESC, COAD, DLBC, KIRC, OV, READ, and THYM, and green for 80%–98% covering BRCA, KICH, KIRP, PRAD, SKCM, TGCT, THCA, UCEC, and UCS, respectively. * is for a *p*-value between 1.0E-10 and 0.05; ** for a *p*-value between 1.0E-010 and 1.0E-20; *** for a *p*-value between 1.0E-20 and 1.0E-30; and **** for a *p*-value <1.0E-40.

The following observations are made: (Wang et al., 2021): upregulated neural genes tend to be involved in the early development of the nervous system such as neuroblast proliferation and neural tube development, while neural functions involved in the late development of the nervous system are generally downregulated, such as sensory and perception related pathways; (Deborde and Wong, 2017); genes involved in neuronal apoptosis are widely observed, revealing that the relevant cancer types have considerable neuron proliferation, knowing that ∼50% of the neurons will undergo apoptosis after proliferation during the normal development of a nervous system (Creeley and Olney, 2010); (Yoneda et al., 2018) numerous neural genes involved in the transport and secretion of neurotransmitters are downregulated, which is consistent with the general observation that cell polarity genes tend to be repressed in cancer (Halaoui and McCaffrey, 2015); and (Jiang et al., 2020) axon guidance genes tend to be upregulated, which is consistent with previous studies as they a) act as tumor suppressors, b) regulate cell migration and apoptosis, and c) control the vascularization of tumors (Klagsbrun and Eichmann, 2005). Their detailed functional roles depend on their receptors (Chédotal et al., 2005). Figure 3A summarizes the levels of these functionalities across the 26 cancer types.

Figure 3B lists all the 11 neural pathways (upper panel, in black) commonly upregulated across the 26 cancer types and all the four downregulated neural pathways (lower panel, in red). The following relationship is observed between the levels of the enriched neural pathways and the survival rates across the 26 cancer types (survival rates collected from the SEER report (Ries et al., 2008)): for the 11 upregulated pathways, the lower the survival rate a cancer has, the more significant the *p*-values that these enriched pathways have, where these pathways are generally found in neuronal communities with strong neuron-neuron interactions. These suggest that more malignant cancers tend to use more complex neural networks, measured using the number of neural genes involved. This is consistent with the previous studies (Magnon et al., 2013; Huang et al., 2014).

#### 3.3.2 Functional analyses of co-expressed neural genes

To elucidate which neural genes tend to work together during a cancer development, we have carried out clustering analyses of co-expressed upregulated neural genes at each stage of each of the 26 cancer types using WGCNA (see METHODS). Supplementary Tables S12, S13 list all the pathways enriched by genes in each cluster in each cancer stage. We note that i) UCS has the highest number of neural gene clusters, at 9, and LGG has the lowest one, at 2; ii) neurogenesis, axonogenesis, gliogenesis, synapse formation, and neural structure formation are the neural functions commonly observed across all 26 cancer types; iii) neuron migration and neuron death tend to be more significantly enriched (with lower *p*-values) in young patients (under 60 years old) of cancers having high survival rates, while early developmental processes such as neural tube development are more significantly enriched in elderly patients (over 60 years old) of cancers with low survival rates; iv) axongenesis is more significantly enriched in female patients of cancer types having low survival rates, but no such a pattern is observed in male patients; v) for early stage patients, cancer types with high survival rates tend to have more significantly enriched neuronal differentiation processes, while for advanced patients, cancers with low survival rates have more significantly enriched early developmental processes, primary neural tube formation and axonogenesis; and vi) the level of endoplasmic reticulum (ER) stress strongly correlates with neural functions in nine cancer types: ACC, GBM, KICH, KIRC, KIRP, LGG, PRAD, and READ. The detailed clusters and their associations are given in Supplementary Table S14 through Supplementary Table S20.

As an example, we show a comparison between two cancer types, THCA and PAAD, in terms of the pathways enriched by their co-expressed neural genes, where the former is a cancer type with the highest survival rate while the latter has the lowest one. We note that while the two cancer types share a few neural functions such as axon guidance, glia activation and synapse formation, PAAD has considerably more neural functions such as myelination, neural potential regulation and neurotransmitter transport related pathways compared to THCA, which has only one unique pathway as given in Supplementary Table S13, suggesting again that cancers with lower survival rates utilize more neurological functions.

#### 3.3.3 Interactions between neural and non-neural genes across different cancer types

**FIGURE 4:**
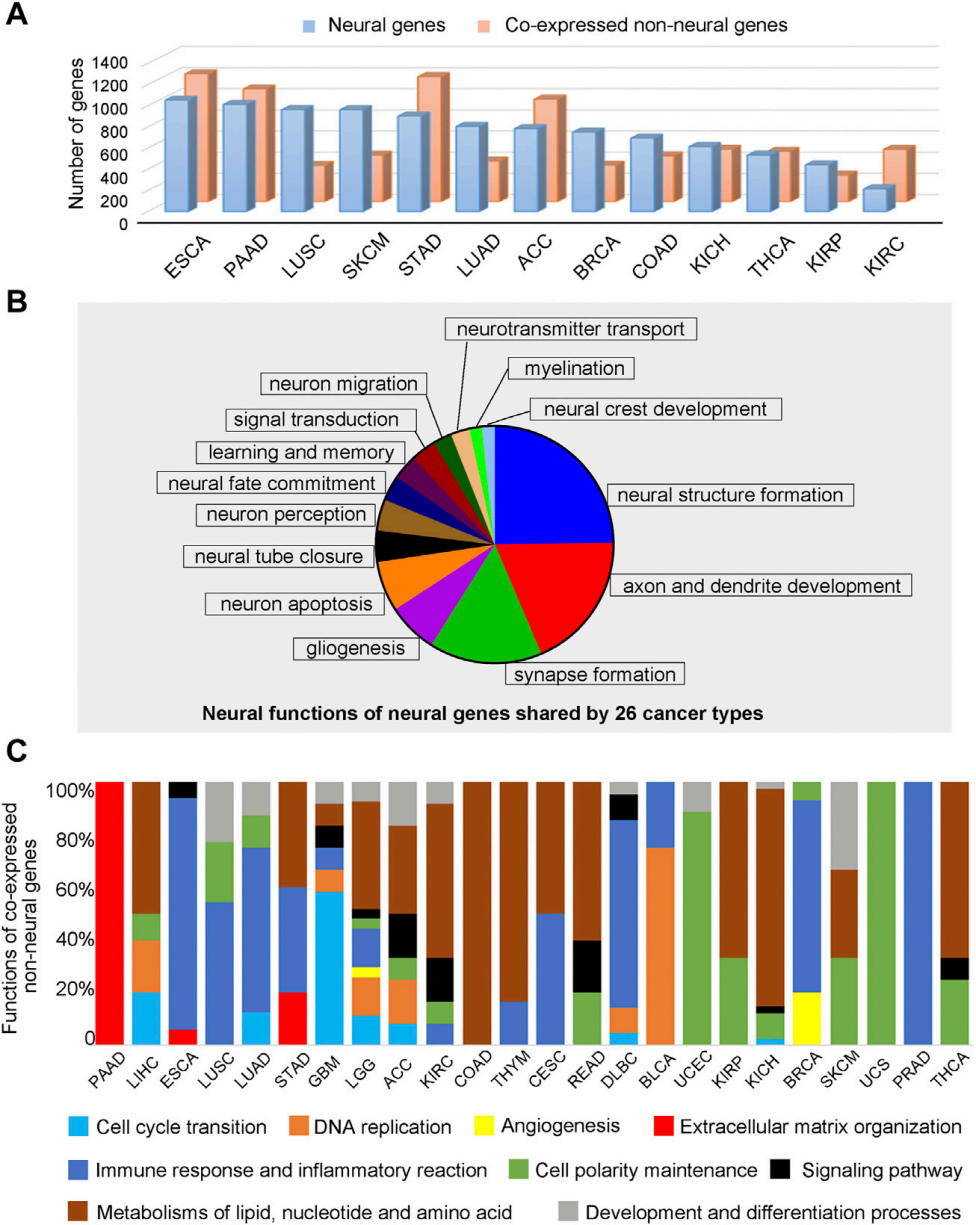
Function analyses of neural genes and co-expressed non-neural genes across 26 cancer types. **(A)** The number of co-expressed neural and non-neural gene pairs across different cancer types. **(B)** Functions of expressed neural genes shared by 26 cancer types. **(C)** Proportions of pathways enriched by co-expressed non-neural genes in TME across different cancer types. Blue is for cell-cycle transition related pathways, orange for DNA replication related pathways, yellow for angiogenesis related pathways, red for extracellular matrix organization related pathways, dark blue for immune response and inflammatory reaction related pathways, green for cell polarity maintenance related pathways, black for signaling pathway related pathways, brown for metabolisms of lipid, nucleotide and amino acid related pathways, and gray is for development and differentiation related pathways.

Overall, we aim to develop a knowledgebase to enable cancer researchers to study the interactive relationships between neural and non-neural functions in cancer tissues. We have analyzed such interactions *via* co-expression analyses in each cancer type. Figure 4A shows the number of such correlated neural and non-neural gene pairs across different cancer types, detailed in Supplementary Tables S21—S22. We note that, among the co-expressed gene pairs, 1,229 neural genes and 24 non-neural genes are shared by the 26 cancer types, indicating that while substantial neural functions are shared by different cancer types, their interacting non-neural partners tend to be different across distinct cancer types.

We have studied the cellular functions by examining the pathways enriched by the 1,229 neural genes, their co-expressed neural genes, and co-expressed non-neural genes in each cancer type, given in Supplementary Table S23 through Supplementary Table S25. We note that the 1,229 genes enrich the following functions, such as neuroblast proliferation, neural tube closure, neurogenesis, neuron migration, axon/dendrite guidance, glial differentiation, myelination, synapse formation, neurotransmitter secretion, neuron apoptosis and neural crest development, which span the entire neural developmental program. In addition, they also partially cover sensory perception, memory and signaling pathways, shown in Figure 4B; Supplementary Table S24.

The co-expressed neural genes enrich, for example, the following pathways listed in the descending order of the number of cancer types sharing them: early development of nervous system differentiation of neurons, axon guidance, synapse formation, neurotransmitter secretion, glial development, and neuron myelination, shared by all 26 cancer types; neural crest development, neuronal action potential, axon regeneration, learning and memory, sensory perception shared by 25 cancer types, neuron apoptosis by 24 cancer types, sympathetic and parasympathetic nerve development by 22 cancer types, and neuroinflammation by 14 cancer types.

Further analyses have revealed that cancers or their bearing organs sharing structural or functional similarities may share common neural functions. We give a few examples here: i) neuronal action potential is most active in ACC, GBM and UCS, while their harboring organs all have secretory functions: the adrenal gland as an endocrine gland can secrete cortical and sex hormones; astrocytes in brain can secrete various molecules to protect the central nervous system when it is damaged (Verkhratsky et al., 2016); and the endometrium can produce secretions in support of egg implantation in uterus. ii) Axon regeneration is most active in LGG, TGCT, and THYM, which all tend to be of low pathological grade (Forst et al., 2014; Baroni et al., 2019). iii) Neuroinflammatory responses are most active in DLBC, ESCA, PRAD, TGCT, and UCS, while their bearing organs share structural similarities, namely, lymph nodes, esophagi, prostates, testicles, and uteri all experience shape-change during their lifetime. The detailed information is given in Supplementary Table S23.

Enriched non-neural pathways shared by all 26 cancer types include immune response, RNA processing and splicing, and transcription and translation-related pathways, such as post-translational modification. In addition, the following functional categories are shared by most of the 26 cancer types: cell-cycle transition, DNA replication, angiogenesis, extra cellular matrix (ECM) assembly, immune and inflammation response, polarity establishment and maintenance, and metabolisms of lipids, nucleotide, and amino acids, summarized in Figure 4C. Furthermore, some non-neural pathways are shared by a varying number of cancer types, listed in the descending order of the number of cancer types sharing them: polarity establishment and maintenance, and metabolisms of lipids, nucleotide, and amino acids are shared by 14 cancer types, respectively; immune and inflammation response are shared by 13 cancer types; and development and growth are shared by 10 cancer types. The details are given in Supplementary Table S25.

We have also examined non-neural functions specific to cancer types having a particular level of five-year survival rates. To do this, we have binned all 26 cancer types into three groups, containing approximately nine cancer types with the highest, intermediate, or the lowest survival rates, respectively, shown in Figure 4C. We have observed: i) cancers with lower survival rates tend to involve more cell cycle-related pathways, immune responses, and ECM assembly pathways; and ii) cancers with higher survival rates tend to have more metabolic pathways and cell polarity-related pathways, such as microtubule-based cilium organization and movement.

Furthermore, our analyses have revealed that the levels of immune and inflammatory responses tend to negatively correlate with axon guidance and neuronal action potential. Table 1 lists the correspondence between nine neurological functions and immune and inflammatory responses in 26 cancer types. Neural functions are ranked in the 26 cancer types according to their activity level (measured using the number of enriched neural pathways) from high to low; and the result shows that the levels of the immune and inflammation responses that strongly correlate with axon guidance and neuronal action potential increase with the reducing levels of neural functions. No such trend is seen in the other seven neurological functions. Hence we speculate that one function played by the neural genes is to suppress the immune and inflammatory responses, which is supported by the literature, namely, i) multiple members of the semaphoring family are known to be anti-inflammatory, such as SEMA6D is a general anti-inflammatory protein (Kang et al., 2018), while SEMA3G-difficient mouse is known to have enhanced production of inflammatory cytokines and SEMA3A suppresses inflammation in atherosclerosis (Nakanishi et al., 2022); ii) acid-sensing ion channels (ASICs), an ion channel in peripheral neurons, are known to suppress the functions of microglia, the predominant immune cell in the central nervous system (Foster et al., 2021); and iii) stimulation of the vagal nerve can alleviate autoimmune diseases, e.g., activated nerves by electrical shock can suppress the immune system (Sundman and Olofsson, 2014).

**TABLE 1:** Effects of nine neural functions on immune responses.

Neural function		Cancer types and the number of the nine neural pathways																																								
Cancer types along with the percentage of immune and inflammatory response functions in all co-expressed non-neural function		ACC		BLCA		BRCA		CESC		COAD		DLBC		ESCA		GBM		KICH		KIRC		KIRP		LGG		LIHC		LUAD		LUSC		OV	PAAD	PRAD	READ	SKCM	STAD	TGCT	THCA	THYM	UCEC	UCS
		0		25		73		50		0		68		88		8		0		8		0		15		0		63		50		0	0	100	0	0	40	0	0	50	0	0
Axon guidance related pathways		20		7		5		8		19		22		11		18		22		25		20		20		12		7		5		12	22	17	18	20	17	23	23	25	6	18
Action potential related pathways		7		1		2		1		5		4		3		7		5		4		3		6		4		1		2		1	5	1	5	0	5	6	5	5	1	7
Neurotransmitter secretion related pathways		15		9		5		7		6		11		12		12		13		10		9		15		7		6		4		1	15	4	12	7	7	14	12	16	5	18
Synapse formation related pathways		47		16		23		16		45		48		20		51		51		42		43		51		27		22		15		26	56	36	42	41	37	56	48	56	29	45
Glial development related pathways		22		14		4		16		16		21		19		22		23		19		17		23		12		12		15		16	17	16	17	21	18	22	18	23	9	18
Axon regeneration related pathways		7		4		7		0		7		8		6		6		6		7		8		9		5		2		7		5	7	6	7	7	6	9	7	9	7	6
neuron myelination related pathways		6		1		1		3		3		6		4		6		5		4		4		6		4		5		1		4	4	5	5	4	4	6	5	6	4	6
Sympathetic and parasympathetic nervous system development related pathways		3		2		0		2		2		3		2		2		3		1		2		1		0		2		2		0	2	1	2	2	2	3	2	3	0	2
Neuroinflammatory responses related pathways		2		0		0		1		1		4		3		1		1		0		0		0		0		0		2		0	2	3	2	0	0	3	0	2	0	4

### 3.4 Data summary and NGC organization

We have developed a database NGC for neural functions at different stages of a cancer development and their interactions with non-neural functions across 26 cancer types for both male and female patients across different ages. The following depicts the key data and information stored in the database as well as the relevant analysis tools in support of utilizing the data and information in the database.

#### 3.4.1 Data in NGC

NGC contains a) the detailed functional information of 4,039 neural genes, collected from NCBI (Sherry et al., 2001), Ensembl (Zerbino et al., 2018), GenBank (Benson et al., 2018), and Gene Ontology (Harris et al., 2004), which is summarized in Supplementary Table S26; b) the expression data of each neural gene in each cancer tissue across 26 cancer types retrieved from TCGA (Weinstein et al., 2013), reprocessed by the UCSC Xena project (Goldman et al., 2020), as well as its expressions in the central and peripheral nervous systems and the vagal nerve throughout the development from embryos to adults, collected from UniProt (Consortium UniProt, 2015) and the mammalian organ development project (Cardoso-Moreira et al., 2019); c) clinical information for each cancer sample, including cancer stage, survival time, patient age, gender and race, all retrieved from TCGA; d) differentially expressed genes in each stage of each of the 26 cancer types as well as across all cancer samples of each cancer type vs. controls; e) pathways enriched by the up- and downregulated genes in (c), respectively; f) strongly co-expressed neural and non-neural gene pairs across samples defined in (c) and pathways enriched by the neural and non-neural genes in the identified gene pairs, respectively; and g) the expression data of individual genes in brain, cerebellum, heart, kidney, liver, ovary, and testis before and after birth are collected from a published study (Cardoso-Moreira et al., 2019). All the sample numbers mentioned above are given in Supplementary Table S27.

#### 3.4.2 Tools supported by NGC

Searching NGC: NGC supports numerous queries to enable a user to retrieve or derive the desired data or information from the database. Table 2 lists the queries currently supported by NGC, which will continue to grow as the project evolves. These queries range from information retrieval about individual genes such as gene functions, the expression profile of a gene at a particular stage of a specified cancer type, the survival rate profiles of a specified cancer type among patients with high vs. low expressions of a given gene, identification of all genes co-expressed with a specified neural gene over a particular set of cancer samples, pathways enriched by given genes, and calculation of co-expressions among specified genes.

**TABLE 2:** Queries currently supported by NGC.

Query	Function
NeuralFunction (X)	Determine if X is a neural gene or neural genes
Function (X)	Retrieve the functional of gene (set) X
Expression (X, C)	Retrieve the expression profile of gene (set) X from cancer type C
Survival (X, C)	Determine if the expressions of gene X has implication to survival of cancer type C
MetastasisRelevant (X, C)	Determine if gene X is statistically associated with metastasis in cancer C
CoexpressedGenes (X, C)	Retrieve genes co-expressed with gene X in cancer C
CoexpressedNerualGene (X, C)	Retrieve all neural genes co-expressed with gene X in cancer C
Interactions (X, C)	Retrieve all TME genes co-expressed with gene X in cancer C

Expression-based analyses: NGC supports a number of analysis tools related to gene expression data, as detailed in Table 3. They currently cover: i) displaying expression profiles of specified genes over a given sample set; ii) displaying differentially expressed genes across a specified set of cancer samples vs. controls; iii) co-expression analyses between a given gene and a specified set of genes over a particular set of samples; and iv) WGCNA-based clustering analyses of a given set of genes over a set of specified samples.

**TABLE 3:** Analysis tools supported by NGC.

Tool	Function
ExpressionProfile (X, C)	Display of the expression profiles of neural gene set X in cancer type C
DifferentialExpression (X, C)	Display of differentially expression profiles of neural gene (set) X in cancer C and control samples
CorrelationAnalysis (X, C)	Pairwise correlation analysis among genes X in cancer type C
WGCNA (X, C)	Clustering analysis of genes X in cancer type C using WGCNA
PathwayEnrichment (X, C)	Pathway enrichment analysis over gene set X in cancer type C
RegressionAnalysis (X, C)	Univariable and multivariable Cox regression analysis of genes X in cancer type C

Enrichment analyses: NGC provides the pathway enrichment analysis over a specified list of genes using the clusterprofiler package (Yu et al., 2012).

Survival analyses: The database supports both the univariate and multivariate Cox regression analyses of the expressions of specified genes over a given set of cancer samples.

#### 3.4.3 User interface

**FIGURE 5:**
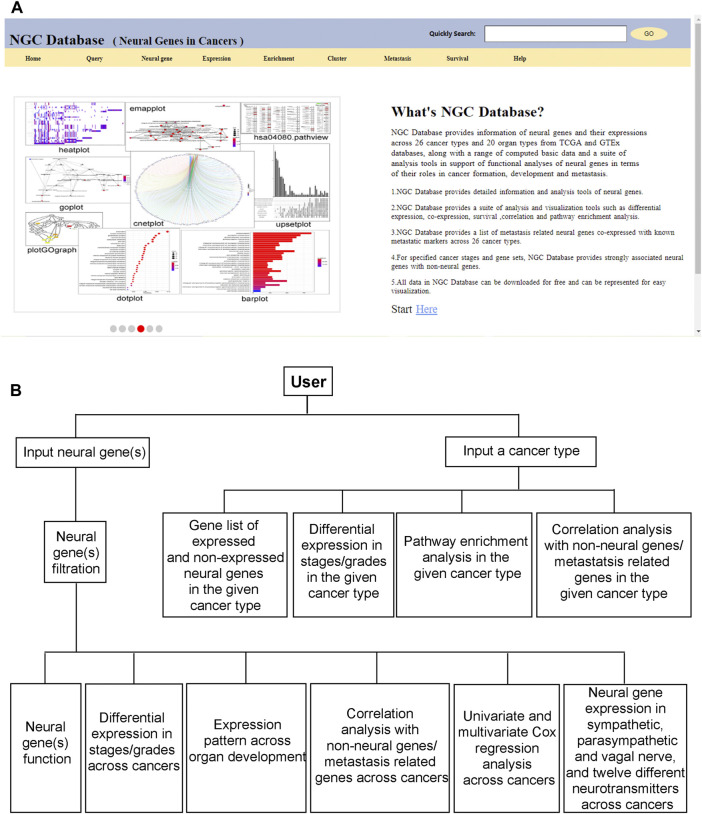
Frontpage and workflow of NGC. **(A)** The frontpage of NGC. **(B)** A workflow of NGC.

NGC provides an intuitive graphics-based user interface to support interactions between a user and the database. All the above functionalities are made available through a panel of clickable buttons in the frontpage of the database, as shown in Figure 5A. Using these buttons, a user can design a gene-centric, cancer type-centric, neural function-centric, or survival centric analysis or conduct comparative analyses of neural functional roles throughout the development of a specific cancer type or a group of related cancer types. Figure 5B shows the overall workflow in utilizing the information and data in NGC.

#### 3.4.4 Database implementation

Django, a python-based web framework, is used to build the web applications in support of user-database interaction. MySQL relational database (version 8.0.16) is employed to store all the information and data presented earlier. HTML, CSS, and JavaScript are used to provide support for web-based display and interactions. A user-friendly web interface is supported by using the Bootstrap (http://getbootstrap.com/) and JQuery (http://jquery.com) extension. R (version 3.6.1) and Python (version 3.7.2) are used for data analyses. NGC Database supports personalized online analyses and visual displays. All search and computational results can be downloaded freely.

## 4 Discussion

We have presented a new database focused on neural functions and their interactions with non-neural functions throughout a cancer development across 26 cancer types. To demonstrate the usefulness of the database, we have provided a number of examples to illustrate how cancer biology questions could be studied from the neural functions’ perspective. To the best of our knowledge, this is the first such database. Compared to the published studies of neural functions in cancer, our study is more systematic in terms of functional roles of neural activities in cancer. Our central theme is that neural functions are induced to help the stressed cells to adapt and survive. Our study is also more comprehensive, covering significantly more neural functions, compared to the published studies (Wang et al., 2021). It is noteworthy that the main functions of the nervous system are to respond to external stimuli, particularly stress-related signals, and to regulate the activities of cell and tissue functions. To execute such roles, the nervous system undergoes complex differentiation to generate neurons and glial cells, and to establish synaptic connections between neurons through neurites to ensure that neuronal action potential will be generated and transmitted properly. Among the major classes of neural functions, we have covered neuron differentiation and neurogenesis, neuron apoptosis, myelination, synapse formation, neurotransmitters, and neurite outgrowth guidance, much broader than what has been covered in the current cancer literature. Furthermore, we have demonstrated how functional associations could be derived among neural functions as well as between neural and non-neural functions, rather than individual neural functions in isolation.

In this study, we have made a number of novel discoveries about the functional roles of neural functions in cancer. For example, we have discovered that different cancers have distinct levels of needs for neurotransmitters, such as cancers with lower survival rates generally need more neurotransmitters. We have also discovered the functional roles by each of the 12 distinct neurotransmitters in cancer metastasis across different cancers.

In the functional roles of neurites, our main discoveries are: i) cancers with poor survival generally involve more neuron-neuron interactions; ii) in female patients, cancers with low survival rates employ more axonogenesis than those having high survival rates; iii) as a patient ages beyond 60, axon guidance will no longer be positively associated with poor survival; and iv) axon-related functions are involved in regulation of the tumor microenvironment (TME). For example, axon regeneration is highly active and strongly correlates with angiogenesis in BRCA.

Another key scientific contribution by our study is that we have established *via* statistical analyses and supported by published studies that the induction of neural functions is predominantly to help the host cells to suppress inflammation and immune responses. In addition, some non-neural pathways are found to strongly co-express with neural pathways in cancer. Examples include that the innate immunity vs. neuroinflammatory response in ESCA; cell cycle-related functions vs. learning, memory, and neuronal action potential in GBM; and angiogenesis vs. axon regeneration in BRCA. These add to the established knowledge that nerves in TME may respond to hypoxia and drive the production of neurotransmitters or neurotrophins for angiogenesis in the tumor environment (Gysler and Drapkin, 2021). These suggest that the emergence of nerves in cancer will not only reduce the inflammation-induced damages, but also provide nutrient supply, helping tumor cells survive.

Overall, we believe that this work will provide a useful information resource for cancer biologists to study the functional roles of the nervous systems through analyzing functional states of neural genes, their interactions with non-neural genes, and pathways they enrich across different cancer types and stages. Such information is organized in a highly modular manner to facilitate relatively easy integration of the stored information as well as novel information syntheses by the user, enabled by the tools provided by the database. We aim to maintain and continue to grow the database on a regular basis, anticipating its utility to the cancer research community. For this reason, we encourage the reader to send us your suggestion for further addition and improvement.

## Data Availability

The datasets presented in this study can be found in online repositories. The names of the repository/repositories and accession number (s) can be found in the article/Supplementary Material.
