# Supplementary material for: Neural functions in cancer: Data analyses and database construction
Source: Front Genet. 2023 Feb 13;14:1062052. doi: 10.3389/fgene.2023.1062052 (PMC9968960; doi:10.3389/fgene.2023.1062052)
Supplement: Supplementary file 1 [file DataSheet1.ZIP › 0112 datasheet1/Supplementary Note.docx]

**Supplementary Note**

This article contains Supplementary Figures S1 to S4, and figure ledges of Supplementary Figure S1 to S4.

This article also contains titles of Supplementary Table S1 to S27.

**
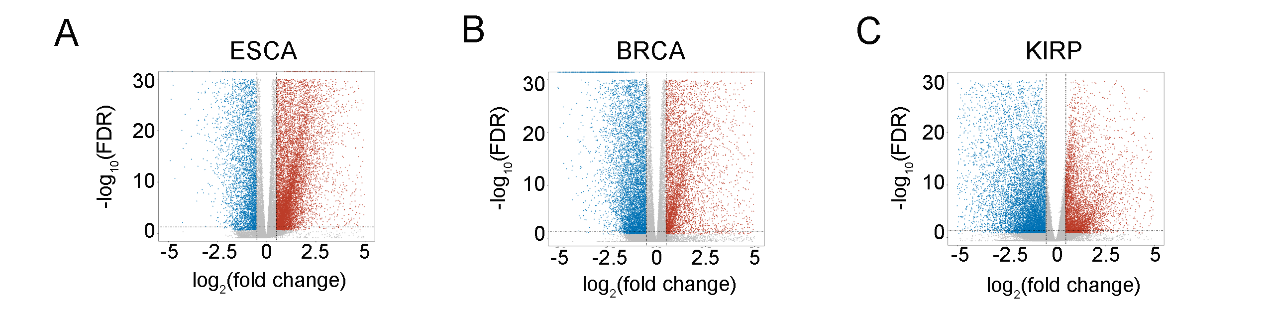
**

**Supplementary Figure S1.** Expression profile of cancers, representing by ESCA, BRCA, and KIRP. **(A)** ESCA, **(B)** BRCA, and **(C)** KIRP, red dots represent up regulated neural genes, blue dots represent down regulated neural genes.

**Supplementary Figure S2.** Line chart of the number of significantly up regulated neural genes at I-IV stages in BRCA, COAD, ESCA, KICH, KIRC, KIRP, LIHC, LUAD, LUSC, SKCM, STAD, THCA. BRCA in blue, COAD in orange, ESCA in light grey, KICH in yellow, KIRC in light blue, KIRP in green, LIHC in Navy blue, LUAD in brown, LUSC in dark grey, SKCM in light Brown, STAD in dark purple, THCA in dark green.

**Supplementary Figure S3.** Line chart of the number of significantly up regulated neural genes at I-IV stages in ACC, BLCA, PAAD, and READ. ACC in blue, BLCA in orange, PAAD in grey, and READ in yellow.

BLCA


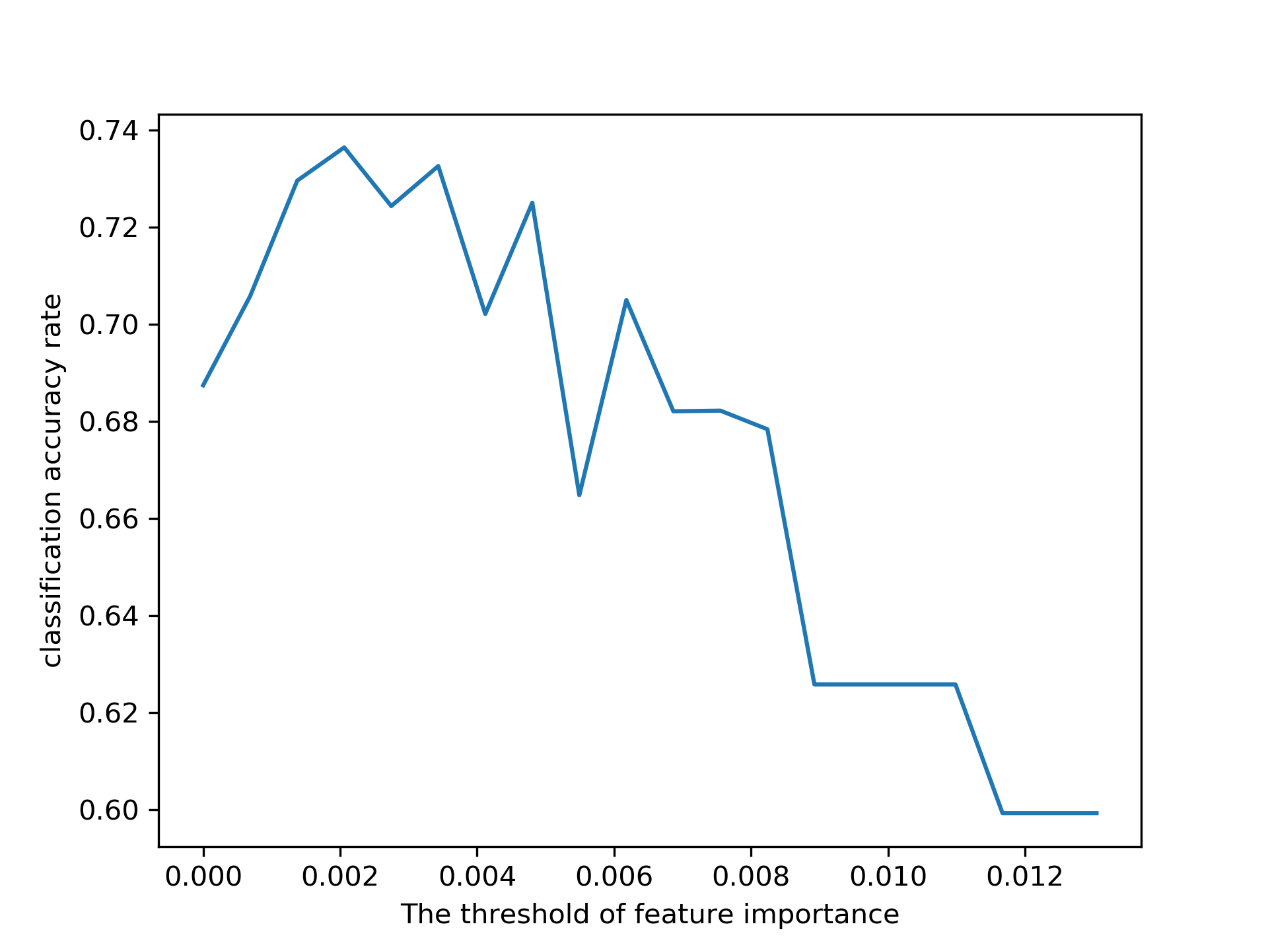


COAD


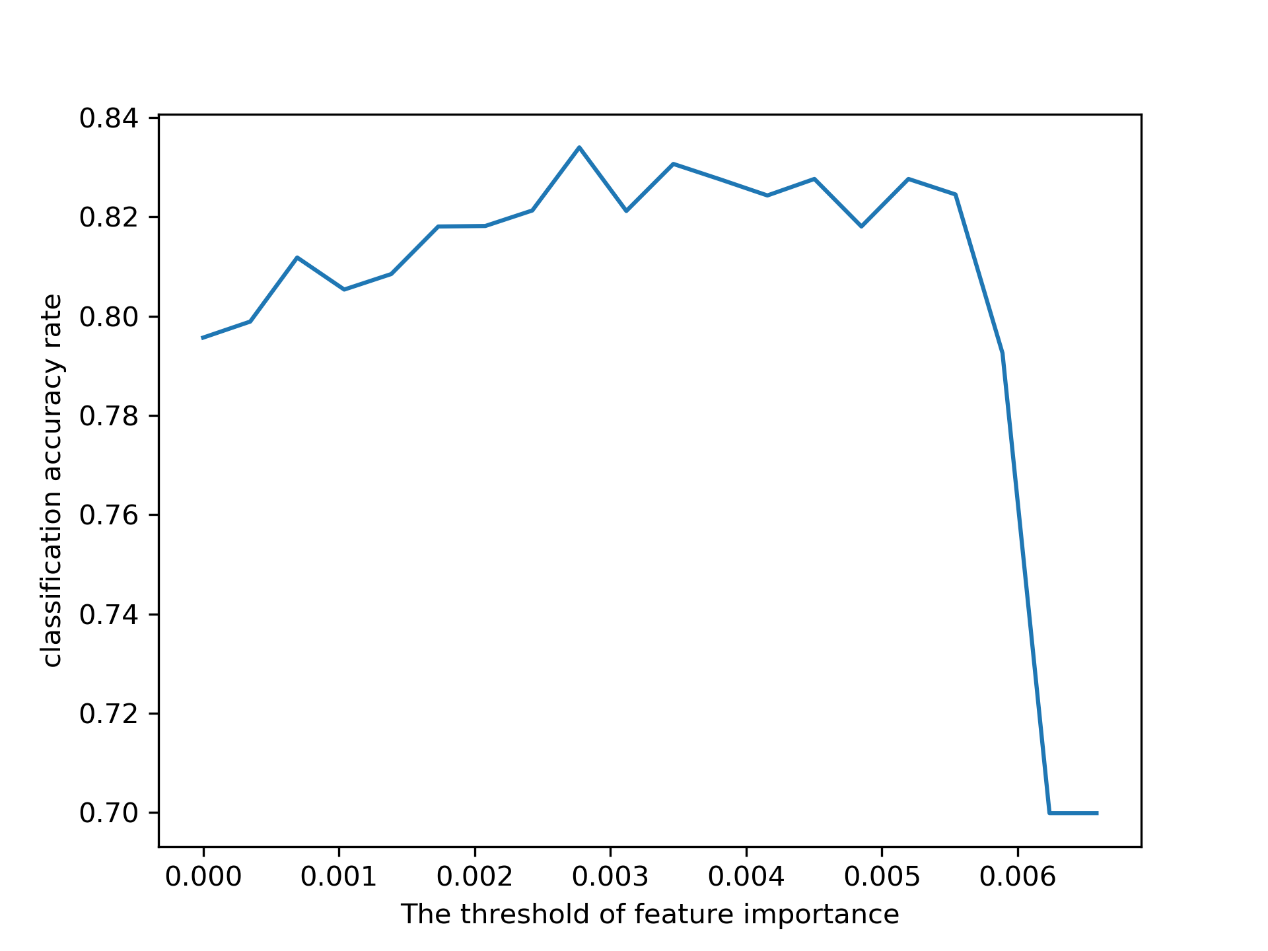


KIRC


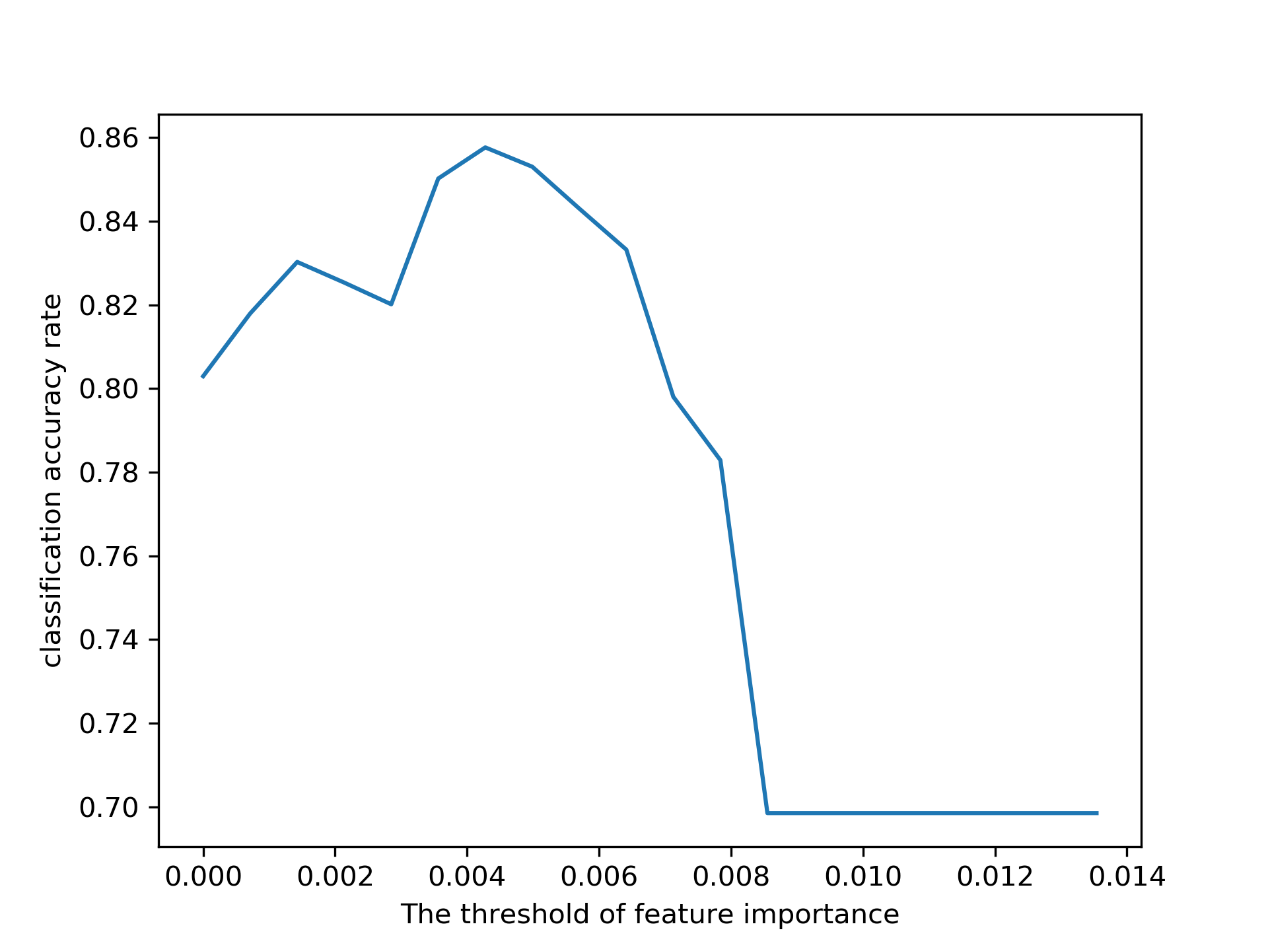


READ


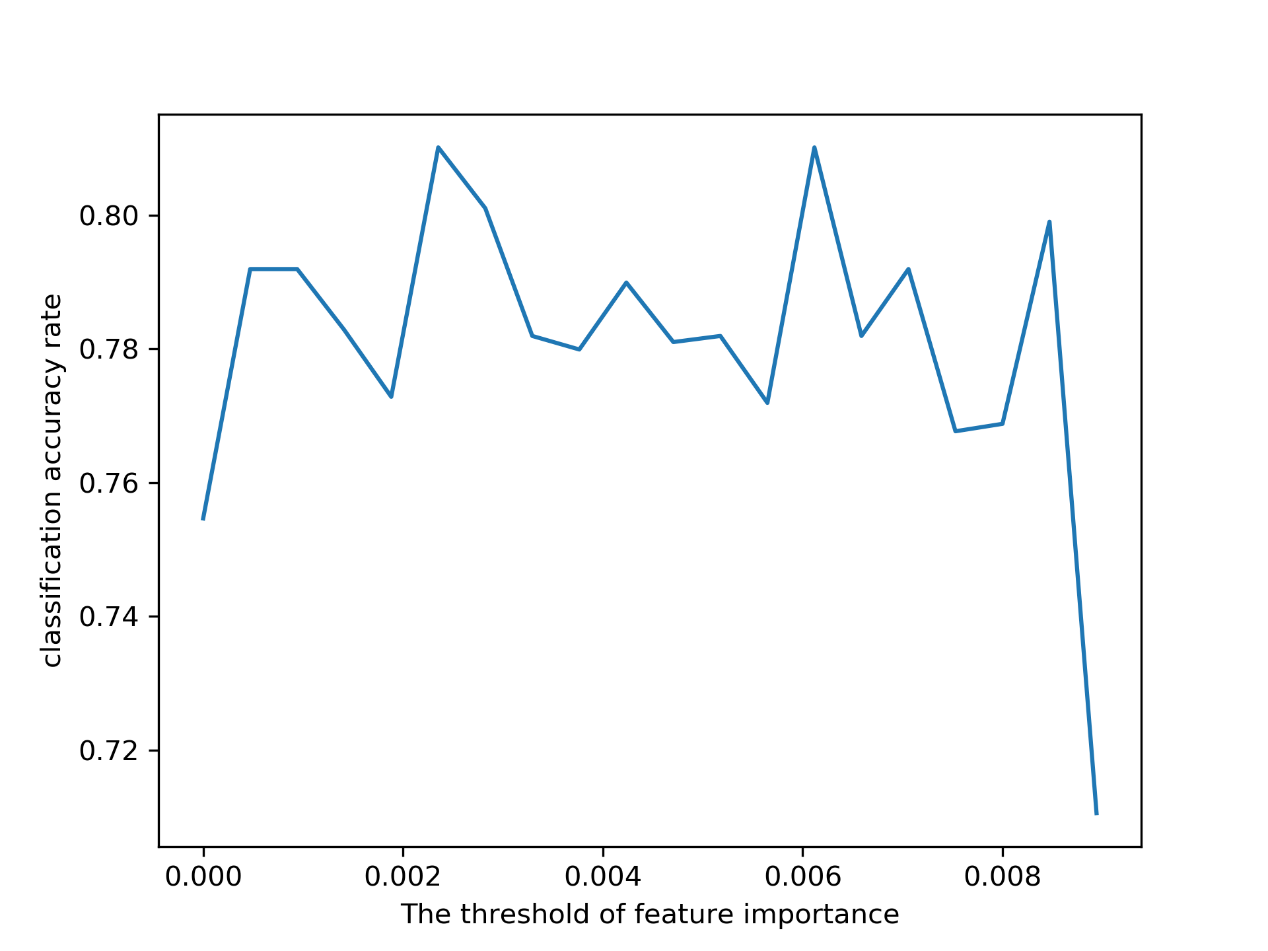


STAD


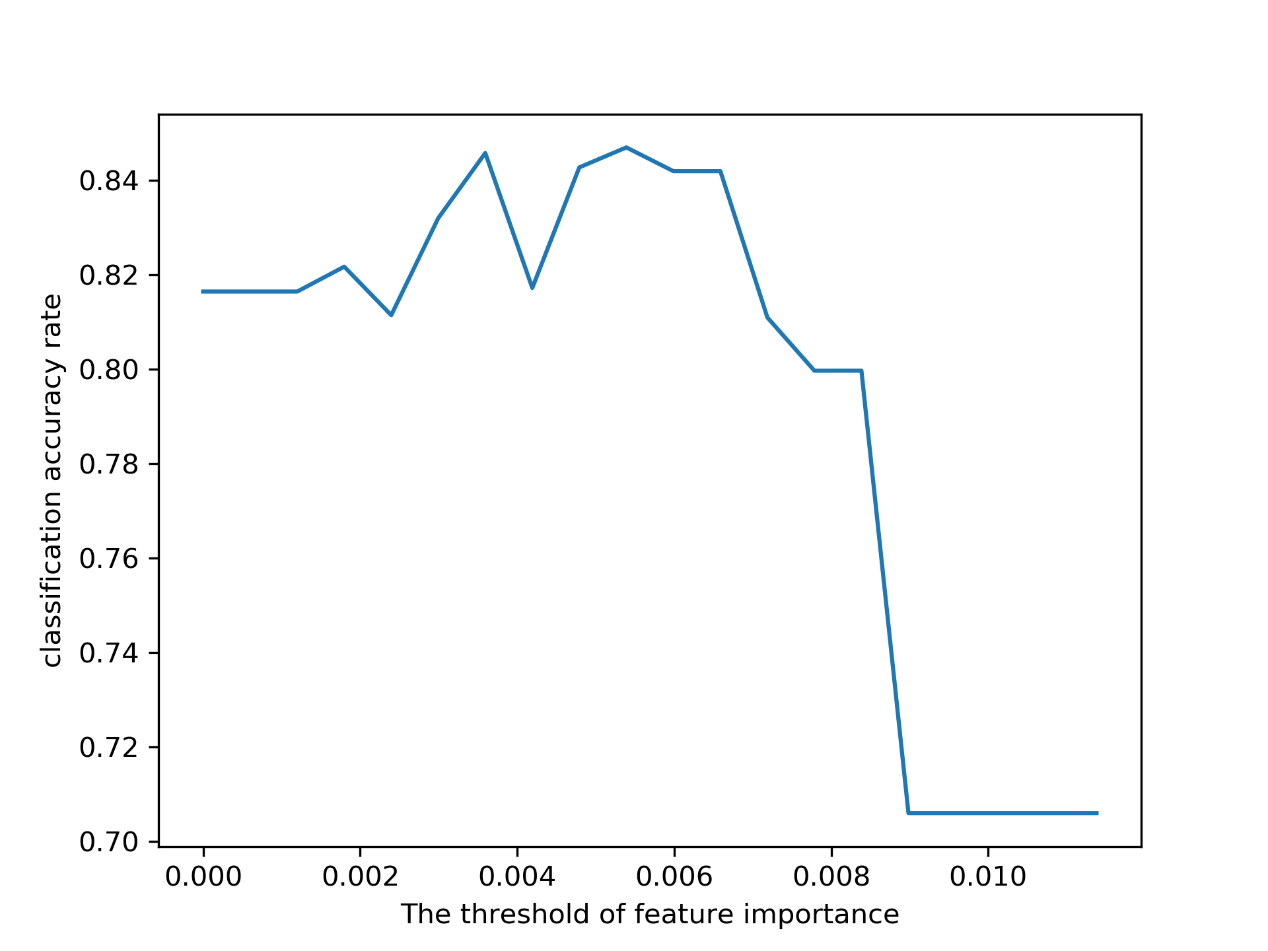


THCA


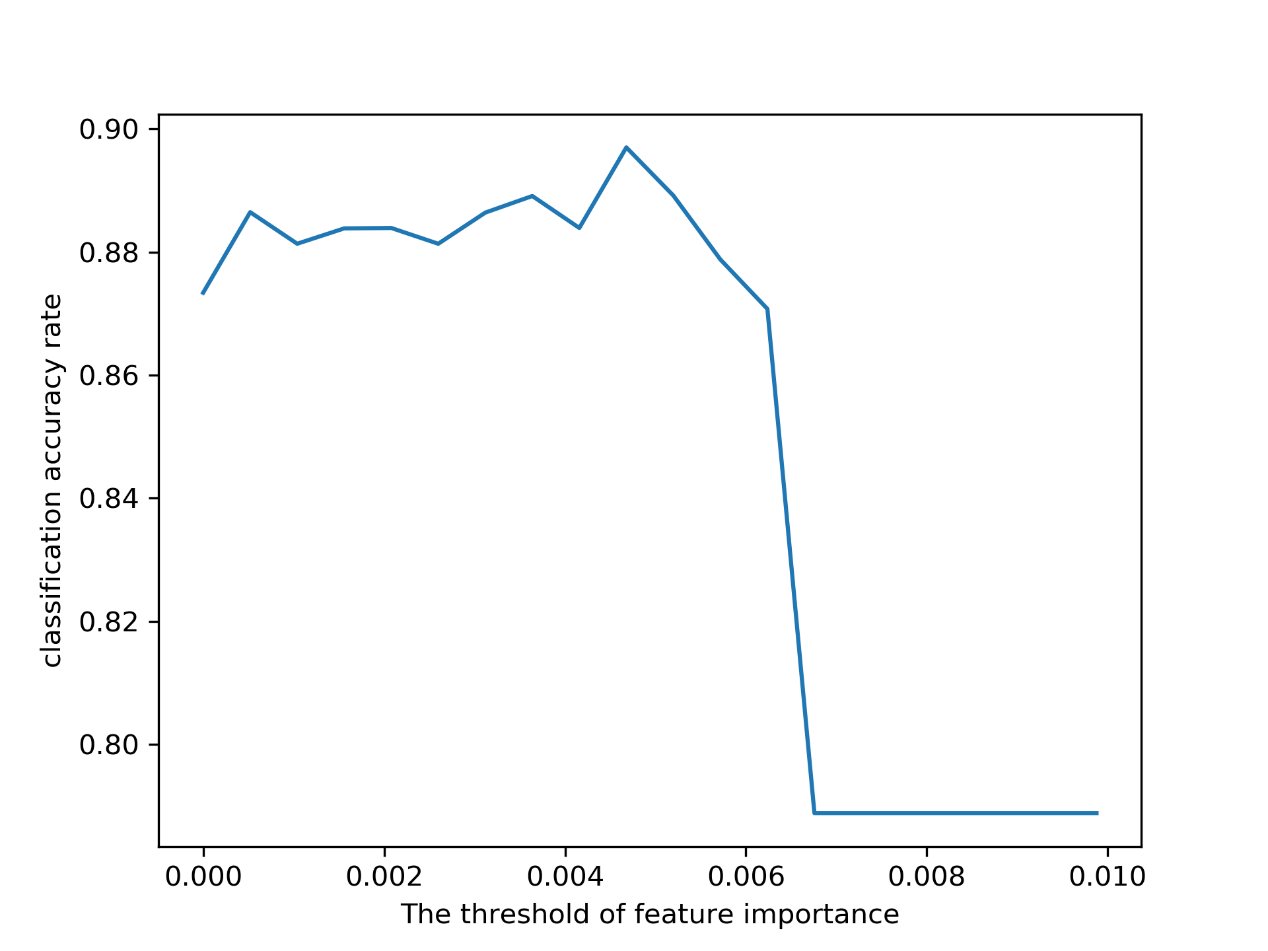
**Supplementary Figure S4.** Line chart of effect of the random forest model in BLCA, COAD, KIRC, READ, STAD, and THCA. The x-axis refers to the threshold of feature importance, the features below the threshold are eliminated, and the y-axis refers to the classification accuracy rate.

**Supplementary Table S1.** Upregulated and downregulated neural genes in each cancer types and their corresponding log2FoldChange, p-value of the differential expression analysis.

**Supplementary Table S2.** The result of univariate Cox regression analysis of up regulated neural genes across 26 cancer types.

**Supplementary Table S3.** The result of univariate Cox regression analysis of down regulated neural genes across 26 cancer types.

**Supplementary Table S4.** The results of LASSO multivariate Cox regression analysis across different cancer types.

**Supplementary Table S5.** The reference of selected neural genes which is critical to the survival of 20 cancer types.

**Supplementary Table S6.** 2091 metastatic genes collected from two databases and a literature by our team.

**Supplementary Table S7.** The MR neural genes and the scores of random forest model across different cancer types.

**Supplementary Table S8.** The number of metastasis-related-neurotransmitters across different cancer types.

**Supplementary Table S9.** The enriched neural pathways of the upregulated neural genes across 26 cancer types.

**Supplementary Table S10.** The enriched neural pathways of the downregulated neural genes across 26 cancer types.

**Supplementary Table S11.** The cluster of neural gene networks of 26 cancer types.

**Supplementary Table S12**. The pathway results of neural gene networks in all clusters of 26 cancer types.

**Supplementary Table S13**. Pathway enrichment results of co-expressed neural genes in younger patients (under 60 years old) across different cancer types.

**Supplementary Table S14.** Pathway enrichment results of co-expressed neural genes in elderly patients (≥ 60 years old) across different cancer types.

**Supplementary Table S15**. Pathway enrichment results of co-expressed neural genes in female patients across different cancer types.

**Supplementary Table S16.** Pathway enrichment results of co-expressed neural genes in male patients across different cancer types.

**Supplementary Table S17**. Pathway enrichment results of co-expressed neural genes in early stage (stage I and stage II) patients across different cancer types.

**Supplementary Table S18.** Pathway enrichment results of co-expressed neural genes in advanced stage (stage III and stage IV) patients across different cancer types.

**Supplementary Table S19**. The result of correlation analysis between co-expressed neural genes in each cluster with ER stress related genes across different cancer types.

**Supplementary Table S20**. Co-expressed result of neural genes across different cancer types.

**Supplementary Table S21.** Co-expressed result of non-neural genes across different cancer types.

**Supplementary Table S22.** Pathway enrichment results of expressed neural genes across different cancer types.

**Supplementary Table S23.** Pathway enrichment results of co-expressed neural genes across different cancer types.

**Supplementary Table S24.** Pathway enrichment results of co-expressed non-neural genes across different cancer types.

**Supplementary Table S25**. 4,039 neural genes that are identified and manually validated based on literature surveys.

**Supplementary Table S26**. Detailed information about the number of cancer and control samples for each cancer type.

**Supplementary Table S27**. Number of cancer samples applied to characteristic neural gene selection during metastasis.
